# Dr. William Wotherspoon Ireland

**Published:** 1909-06-05

**Authors:** 


The death is announced of Dr. William Wotherspoon Ireland, the distinguished Scottish alienist, at his residence at Musselburgh. Dr. Ireland was a member of the Psychiatric Society of St. Petersburg, of the New York Medico-Legal Society, and of the Società Freniatrica Italiana. For many years he held the post of medical superintendent of the Scottish National Institution for the Education of Imbecile Children, and medical officer of Miss Murray's Institution for Girls at Prestonpans. Dr. Ireland was at one time attached to the Indian Army, and was the author of a history of the siege of Delhi.

The Duke of Northumberland, President of Middlesex Hospital, presided over the last meeting of the Court of Governors, when Major-General Lord Cheylesmore called attention to the exceptionally straitened circumstances in which the hospital finds itself. Ten or twelve subscribers have written that, owing to the Budget proposals, they would not be able to subscribe any longer. Mr. Pearce Gould said that the medical school, in view of the increasing employment of radium as a therapeutic agent, needs a larger stock for use upon cancer patients, since there is no doubt that it has a power over certain growths.

